# Identification and Physicochemical Characteristics of Temozolomide Process-Related Impurities

**DOI:** 10.3390/molecules181215344

**Published:** 2013-12-11

**Authors:** Marta Łaszcz, Marek Kubiszewski, Łukasz Jedynak, Monika Kaczmarska, Łukasz Kaczmarek, Wojciech Łuniewski, Krzysztof Gabarski, Anna Witkowska, Krzysztof Kuziak, Maura Malińska

**Affiliations:** 1 Pharmaceutical Research Institute (PRI), Warszawa 01-793, Poland; E-Mails: m.kubiszewski@ifarm.eu (M.K.); l.jedynak@ifarm.eu (Ł.J.); m.kaczmarska@ifarm.eu (M.K.); l.kaczmarek@ifarm.eu (Ł.K.); w.luniewski@ifarm.eu (W.Ł.); k.gabarski@ifarm.eu (K.G.); a.witkowska@ifarm.eu (A.W.); k.kuziak@ifarm.eu (K.K.); 2 Faculty of Chemistry, University of Warsaw, Warszawa 02-093, Poland; E-Mail: mauramalinka@gmail.com

**Keywords:** temozolomide intermediate, X-ray crystallography, nuclear magnetic resonance, high-performance liquid chromatography

## Abstract

In this article the crystal structures of the starting material **TZ-5** and the key intermediate **TZ-6** of temozolomide (**TZ-7**), an anticancer therapeutic agent, are presented, together with their spectroscopic and thermal characteristics. Both compounds crystallize in the triclinic P-1 space group. X-ray crystallography studies proved that the compound **TZ-6** exists as a monohydrate. A complete structural assignment was obtained for the signals in the ^1^H-, ^13^C- and ^15^N-nuclear magnetic resonance spectra and the structures were confirmed by Fourier-Transform infrared and Raman spectroscopy. The article describes the importance of the high purity of **TZ-6** during the small-scale plant production of **TZ-7** in a desired polymorphic form III with the purity higher than 99.50%, according to an HPLC method.

## 1. Introduction

A complete physicochemical characterization has recently become required by both the U.S. Food and Drug Administration (FDA) [1] and by the European Medicine Agency (EMA) [2], not only for active pharmaceutical ingredients (API), but also for all key synthetic intermediates. A well documented impurity profile, the identification and physicochemical characteristic of a starting material and intermediate products are necessary for an API registration.

Temozolomide (**TZ-7**, 3,4-dihydro-3-methyl-4-oxoimidazo[5,1-d]-as-tetrazine-8-carboxamide) is a cytotoxic antitumor prodrug against malignant brain tumours. At physiological pH temozolomide undergoes decomposition to the active compound MTIC (3-methyl-(triazen-1-yl)imidazole-4-carboxamide) [3,4,5]. The drug (brand names: Temodar, Temodal and Temcad^®^), originally developed by Schering-Plough Corp., first bacame available in the U.S. in August 1999 [6]. X-ray crystallography studies of temozolomide have been described by Lowe *et al*. [7]. The crystal structures of temozolomide polymorphs, as well as its cocrystals with 4,4'-bipyridine-*N,N*'-dioxide have been reported by Jagadeesh Babu *et al*. [8].

In this article the complete physicochemical characteristics of temozolomide intermediate and starting material have been determined by the following methods: ^1^H-, ^13^C- and ^15^N-nuclear magnetic resonance (NMR), infrared (IR) and Raman spectroscopy, as well as by thermal analyses. Single crystals of both compounds were obtained and their X-ray structures were solved. Furthermore, high-performance liquid chromatography (HPLC) methods for the purity determination of **TZ-5** and **TZ-6** were evaluated.

## 2. Results and Discussion

### 2.1. Structure Elucidation by NMR, IR and Raman Spectroscopy

#### 2.1.1. NMR Spectroscopy

All signals in the ^1^H-, ^13^C- and ^15^N-NMR spectra of temozolomide (**TZ-7**) and key intermediates **TZ-6** and **TZ-5** were assigned to the respective atoms using one- and two-dimensional techniques (g-HSQC and g-HMBC (^1^H-^13^C and ^1^H-^15^N-NMR, Table 1).

The comparison of **TZ-5**, **TZ-6** and **TZ-7** spectra shows that these compounds have a very similar skeletal systems, especially the **TZ-7** and **TZ-6** pair. Both molecules have the same imidazotetrazine ring in the structure. Their spectra differ only in the region of the amide group. In the imidazole ring the differences between the corresponding values of chemical shifts are usually not more than 0.5 ppm. Only for the nitrogen at position 8 is the difference bigger, *i*.*e*., 1.88 ppm. The *tert*-butyl group substitution in the **TZ-7** molecule also increases the shielding of the carbonyl carbon in position 10 (the chemical shift changes from 161.51 ppm to 158.93 ppm). Unfortunately, in our study we did not determine any chemical shift values for the nitrogen atoms in position 2 of the molecules **TZ-7** and **TZ-6**. For the compound **TZ-7** this value was measured using an isotopically labeled compound in the work of Wheelhouse *et al*. [9]. In that article the chemical shift of the nitrogen atom 2 is −33.99 ppm for **TZ-7** and a similar value was expected for **TZ-6**.

**Table 1:** Signal assignment for **TZ-7**, **TZ-6** and **TZ-5** in ^1^H- ^13^C- and ^15^N-NMR spectra, (in DMSO-d_6_ solution).

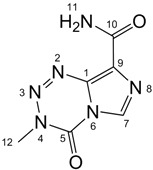 TZ-7			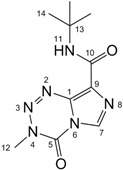 TZ-6			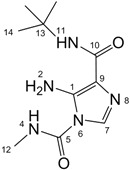 TZ-5		
Position	^1^H (ppm)	^13^C/^15^N (ppm)	Position	^1^H (ppm)	^13^C/^15^N (ppm)	Position	^1^H (ppm)	^13^C/^15^N (ppm)
**1**	−	134.58	**1**	−	134.21	**1**	−	111.67
**2**	−	not obtained	**2**	−	not obtained	**2**	s, 2H, 6.33	−327.03
**3**	−	19.59	**3**	−	19.42	−	−	−
**4**	−	−180.37	**4**	−	−180.82	**4**	q, 1H, 8.44	−293.78
**5**	−	139.18	**5**	−	139.15	**5**	−	150.68
**6**	−	−199.86	**6**	−	−200.03	**6**	−	−202.82
**7**	s, 1H, 8.81	128.36	**7**	s, 1H, 8.80	128.22	**7**	s, 1H, 7.61	125.90
**8**	−	−106.03	**8**	−	−107.91	**8**	−	−125.92
**9**	−	130.50	**9**	−	130.87	**9**	−	142.74
**10**	−	161.51	**10**	−	158.93	**10**	−	163.84
**11**	s, 1H, 7.67; s, 1H, 7.79	−274.71	**11**	s, 1H, 7.55	−246.00	**11**	s, 1H, 6.59	−255.99
**12**	s, 3H, 3.87	36.13	**12**	s, 3H, 3.87	36.14	**12**	s, 3H, 2.79	26.56
−	−	−	**13**	−	50.77	**13**	−	49.80
−	−	−	**14**	s, 9H, 1.42	28.49	**14**	s, 9H, 1.36	28.94

#### 2.1.2. IR and Raman Spectroscopy

**Figure 1:**
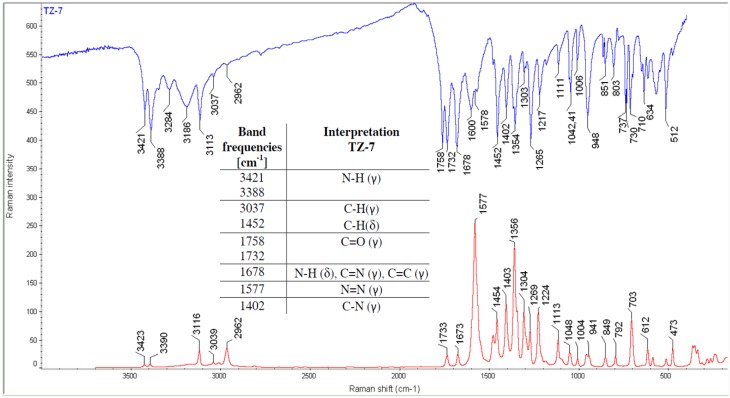
IR and Raman spectra of **TZ-7**.

The structures of all the compounds were further confirmed by IR and Raman spectroscopy. Three characteristic bands at: 1678, 730 and 710 cm^−1^ [10] proved the presence of the form III in the **TZ-7** sample (Figure 1). A doublet at 3421 and 3388 cm^−1^ is presumed to have come from the primary amide N-H stretching vibrations (γ). At 1758 and 1732 cm^−1^ the first amide band (C=O (γ)) from the primary and tertiary amide is manifested and the band at 1678 cm^−1^ can come from the second amide band (N-H bending vibrations (δ)) as well as from C=N (γ) and C=C (γ) vibrations. It is supposed that the most intensive Raman band at 1577 cm^−1^ can come from the N=N (γ) vibration.

**Figure 2:**
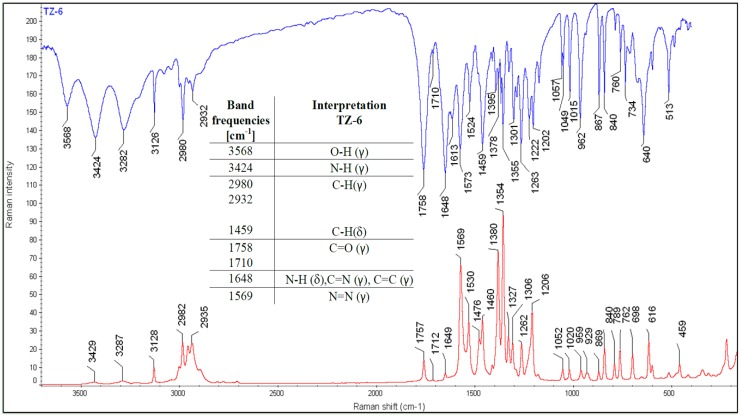
IR and Raman spectra of **TZ-6**.

The band at 3568 cm^−1^ is visible only in the IR spectrum of **TZ-6** (Figure 2). This band is not visible in the Raman spectrum. It is supposed that this band can indicate the presence of a water molecule incorporated into the crystal structure of **TZ-6**. The band at 3424 cm^−1^ can indicate the presence of the secondary amide N-H (γ) vibration. At 1758 and 1710 cm^−1^ the first amide band from the secondary and tertiary amide is manifested and the band at 1648 cm^−1^ can come from the second amide band as well as from C=N (γ) and C=C (γ) vibrations. It is supposed that the band at 1569 cm^−1^ represents the N=N (γ) vibration.

**Figure 3:**
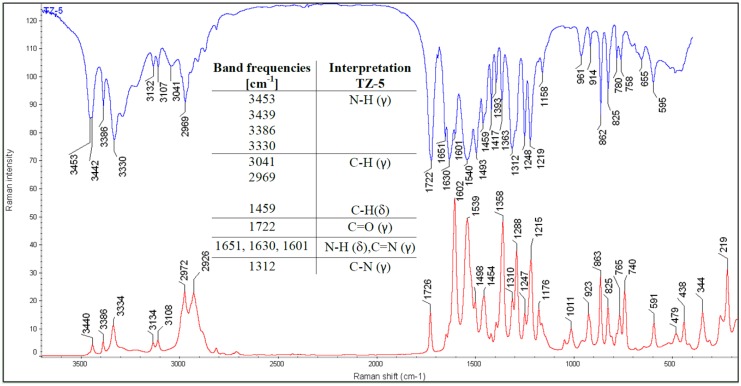
IR and Raman spectra of **TZ-5**.

Presumably, in **TZ-5** (Figure 3) the bands from 3453 to 3330 cm^−1^ manifest the presence of the N-H (γ) vibration form the amide and amine groups as well. The most intensive band in the IR spectra, at 1721 cm^−1^ indicates the C=O (γ) vibration. The bands at 1651, 1630 and 1601 cm^−1^ can come from the N-H (δ) and C=N (γ) vibrations.

#### 2.1.3. X-Ray Cyrstallography Studies

**Figure 4:**
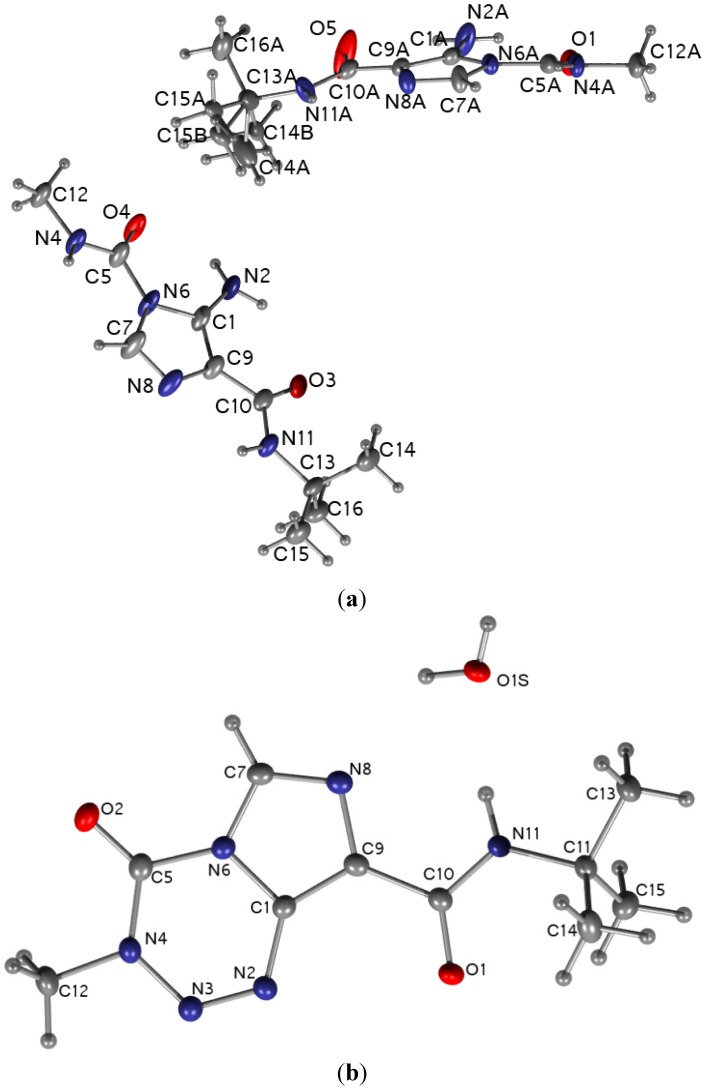
Molecular structure and atomic labelling of **TZ-5** (**a**) and **TZ-6** (**b**). Thermal displacement ellipsoids are drawn at the 50% probability level.

The crystal structure of temozolomide has been previously reported [7,8]. **TZ-5** crystallises in the triclinic P-1 space group with two molecules in the asymmetric unit, whereas **TZ-6** crystallises as a hydrate with one molecule of water. The molecular structures of **TZ-5** and **TZ-6** are depicted with the numbering scheme in Figure 4.

The differences in the corresponding structural parameters of the two molecules in the asymmetric unit of the **TZ-5** crystal structure are within the level of errors (less than 3σ). The only significant difference between these molecules is the disorder of the side chain present in one of them. Therefore, the numerical values of interatomic distances will be reported only for one molecule. The **TZ-5** molecules are planar and form an intramolecular hydrogen bond between the amine group and the carboxylic oxygen atom. The distances between the acceptor and donor of the hydrogen bond are equal to: 2.91(1)Å and 2.90(1)Å for O(4)…N2 and O(3)…N2, respectively. The planarity of the ring and amide bond is reserved in the **TZ-6** molecules where the intramolecular hydrogen bonds are impossible. However, **TZ-6** crystallises with one molecule of water per molecule of **TZ-6** and the water forms four hydrogen bonds with the precursor (N11…O1S 2.903(1)Å, O1S…N8 2.887(1)Å, O1S…N2*_x_*_,*y+1*,*z*_ 3.067(1)Å, O1S…O1*_x_*_,*y+1*,*z*_ 2.719(1)Å).

**Figure 5:**
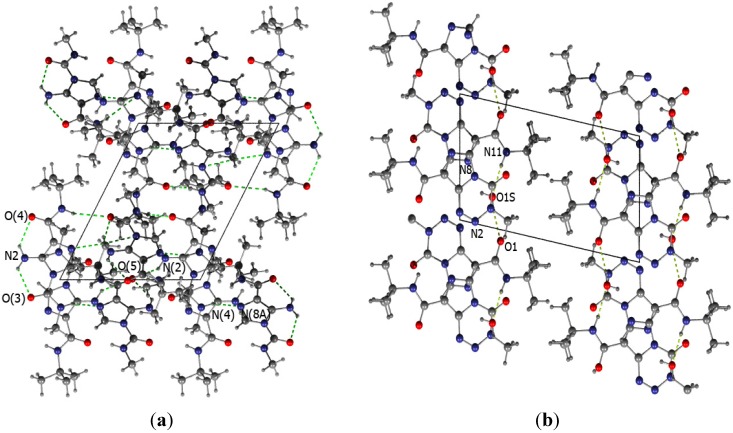
The view of crystal packing of **TZ-5** along the *y* axis (**a**) and crystal packing of **TZ-6** along the *x* axis (**b**). The hydrogen bond is marked by a green, dashed line.

Both structures reveal a similar layered packing scheme with easily distinguished hydrophilic and hydrophobic parts with the supremacy of hydrogen bonds and dispersive interaction, correspondingly (see Figure 5).

The packing of **TZ-5** molecules is based on four hydrogen bonds, *i*.*e*., two for one molecule from the asymmetric unit (N4…N8A*_-x_*_,*-y+1*,*-z*_ 2.89(1)Å, N2…O5*_-x+1_*_,*-y+1*,*-z*_ 2.91(1)Å). The hydrogen bonds between the **TZ-6** molecule and water are responsible for the formation of the layered structure with alternating **TZ-6** moieties and water molecules (see Figure 5). In the second direction, the molecules are hold together by π…π stacking between the heteroaromatic rings. For both reported structures, the interactions between layers are dispersive. These are mostly the H…H interactions between side chains of molecules. The **TZ-7** molecule [7,8] does not have a bulky *tert*-butyl substituent, and its crystal structure exhibits a more effective packing. In this case, the hydrogen bonds govern the packing in the crystal lattice in all directions.

#### 2.1.4. Thermoanalitycal Studies

**Figure 6:**
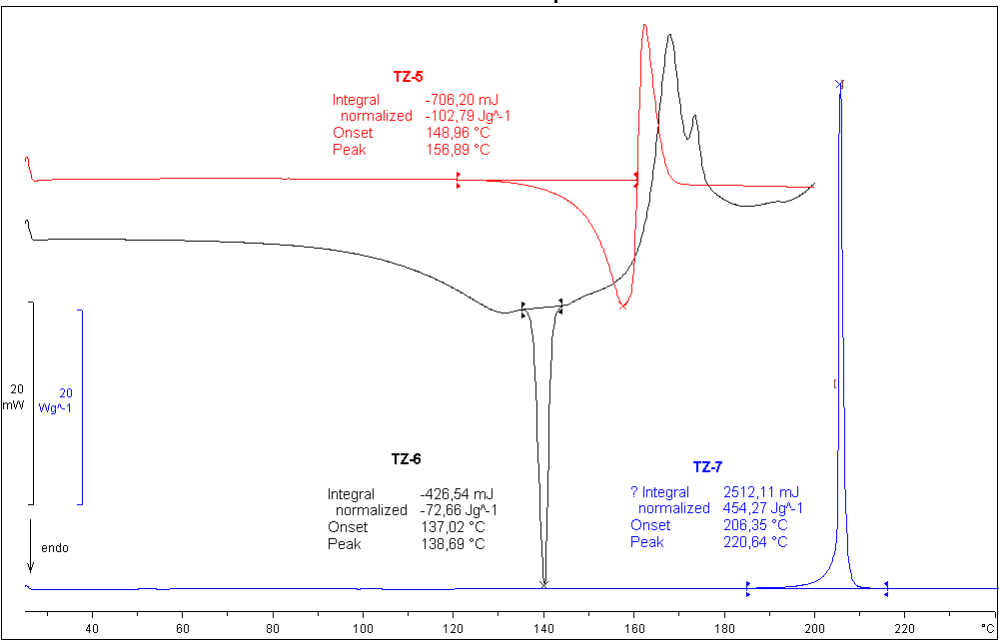
DSC curves of **TZ-5**, **TZ-6** and **TZ-7**.

Figure 6 shows the DSC curves of **TZ-5**, **TZ-6** and **TZ-7**. The DSC curve of **TZ-5** is characterized by one exothermal signal from the substance decomposition, followed by an endothermic melting peak at 148.96 °C.

The endothermic melting effect of **TZ-6** is at 137.02 °C, in the range of decreasing baseline from 100 to 130 °C. A thermogravimetric analysis proved a mass loss of 6.70% in this range. This value agrees well with water content in the sample and theoretical water content for a monohydrate (6.71%). Endothermic effects visible in the temperature range from 160 to 180 °C come from the decomposition of the substance. The DSC curve of temozolomide only exhibits the exothermic decomposition effect at 206.35 °C. The mass loss value calculated from the thermogravimetric curve in the range of this effect is above 50%.

## 3. Experimental

### 3.1. Synthesis

#### 3.1.1. Synthesis of 5-amino-*N*-(*tert*-butyl)-3-(methylcarbamoyl)imidazole-4-carboxamide (**TZ-5**) [11] (Scheme 1).

**Scheme 1:**
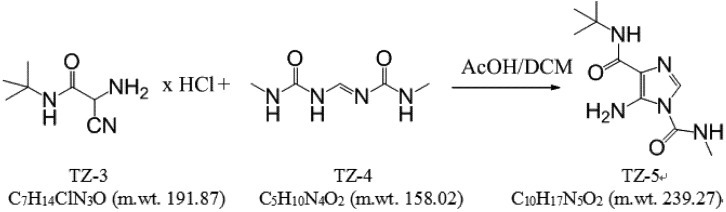
The snthesis of **TZ-5**.

The mixture of the compounds **TZ-3** [12] (96.0 g, 0.5 mol) and **TZ-4** [13]: (95.0 g, 0.6 mol) was diluted with dichloromethane (1.0 L), then acetic acid (100%, 5 mL) was added. The whole mixture was stirred at ambient temperature for 1 h, refluxed for an 1 h and then cooled to ambient temperature. The mixture was neutralized with aqueous potassium carbonate (pH 4–5), the organic layer was separated and the aqueous one extracted with dichloromethane (2.0 L). The combined extracts were evaporated *in vacuo* to approx. 20% of their volume, the precipitate collected and washed with dichloromethane (75 mL). The filtrate was evaporated to approx. 50% of volume and refrigerated at −10 °C for 16 h. The precipitate was collected and washed with dichloromethane (25 mL). The combined precipitates were dried *in vacuo* at 30 °C to afford 87 g (73%) of the compound **TZ-5** in the form of yellow crystals. Elementary analysis: 50.07% (C), 7.18% (H), 29.06% (N). ESI-MS: calcd. for (M+H)^+^: 239.14, found 239.14.

#### 3.1.2. Synthesis of *N*-(*tert*-butyl)-3-methyl-4-oxo-3,4-dihydroimidazo[5,1-d]-1,2,3,5-tetrazine-8-carboxamide (**TZ-6**) [11] (Scheme 2).

The compound **TZ-5** (50.0 g, 0.21 mol) was added at 10 °C to the stirred solution of lithium chloride hydrate (378 g) and acetic acid (24 mL) in water (1 L). The mixture was stirred for 30 min and cooled to 4 °C, then sodium nitrite (22 g, 0.32 mol) was added in one portion. The whole mixture was stirred for 1 h at 4–5 °C and for next 18 h at ambient temperature, then diluted with dichloromethane (1.0 L) and stirred vigorously for 10 min. The organic layer was separated and the aqueous one extracted twice with 500 mL and 250 mL portions of dichloromethane.

**Scheme 2:**
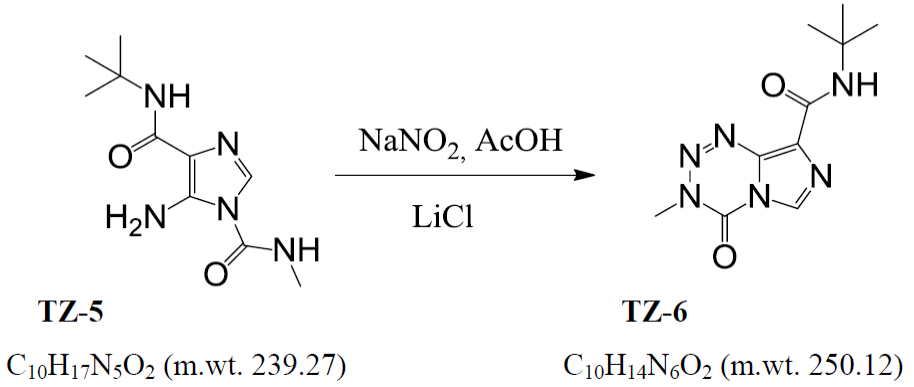
The synthesis of **TZ-6**.

The combined extracts were then washed with 10% aqueous sodium dithionite (1.0 L) and evaporated to dryness *in vacuo* at temperature not exceeding 25 °C. The crude product was suspended in the mixture of 2-propanol–water–36% hydrochloric acid (1,000 mL–225 mL–25 mL) and refluxed for 20 min. After filtration the solution was left at ambient temperature for 24 hrs, the precipitate collected, washed with 80% aqueous 2-propanol (125 mL) and dried *in vacuo* at ambient temperature to afford the compound **TZ-6** of HPLC 99.9% + purity in the yield 28.5 g (54%). Elementary analysis: 44.77% (C), 6.02% (H), 31.23% (N). ESI-MS: calcd. for (M+H)^+^: 250.12, found 250.12.

#### 3.1.3. Synthesis of 3,4-dihydro-3-methyl-4-oxoimidazo[5,1-d]-as-tetrazine-8-carboxamide (**TZ-7**) [11] (Scheme 3).

**Scheme 3:**
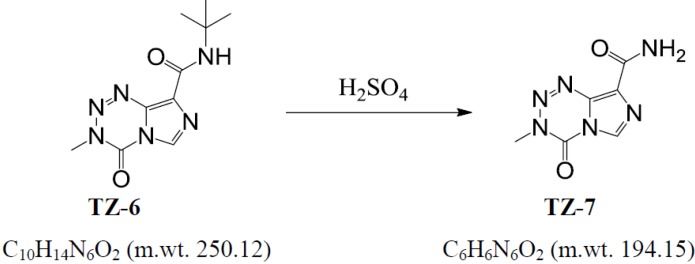
The synthesis of **TZ-7**.

The compound **TZ-6** (25,0 g, 0,10 mol) was added portionwise to stirred sulfuric acid (d 1.8, 70 mL) at 15–25 °C. The whole mixture was stirred at ambient temperature for 3 h and then poured into 96% ethanol (800 mL) at 0 °C. The suspension was stirred for 30 min. at 0 °C, the precipitate collected, washed with cold ethanol (50 mL) and dried on air for 16 h. The obtained crude **TZ-7** was dissolved in the refluxed mixture of acetone (450 mL), water (150 mL) and acetic acid (38 mL), the solution was filtered, the filtrate refluxed again, cooled to ambient temperature and stirred for 4 h. The precipitate was then collected, washed with acetone/water 3:1 (80 mL) and dried *in vacuo* for 16 h at 25 °C to afford 10.1 g (52%) of pharmaceutically pure **TZ-7**. Elementary analysis: 36.99% (C), 3.00% (H), 43.02% (N). ESI-MS: calcd. for (M+H)^+^: 194.06, found 194.06.

### 3.2. Techniques

#### 3.2.1. X-Ray Crystallography

The deposition numbers CCDC 806024 (**TZ-5**) and CCDC 805454 (**TZ-6**) contain the supplementary crystallographic data. These data can be obtained free of charge via www.ccdc.cam.ac.uk/conts/retrieving.htmL (or from the CCDC, 12 Union Road, Cambridge CB2 1EZ, UK; fax: +44-1223-336033; e-mail: deposit@ccdc.cam.ac.uk).

The data of **TZ-5** and **TZ-6** were collected using the Bruker KAPPA APEXII ULTRA diffractometer controlled by the APEXII software [14], equipped with the MoKα rotating anode X-ray source (λ = 0.71073 Å, 50.0 kV, 22.0 mA) monochromatized by multi-layer optics and APEX-II CCD detector. The data collection was carried out at 100 K using the Oxford Cryostream cooling device. Indexing, integration and initial scaling were performed with the SAINT [15] and SADABS [16] software (Bruker AXS, Madison, WI, USA, 2008). The structures were solved by direct methods using the SHELXS-97 [17] program and refined with the SHELXL-97 [15]. A multi-scan absorption correction has been applied in the scaling procedure. Crystal structure parameters are reported in Table 2 for the corresponding structures. 

**Table 2:** Crystal structure parameters for **TZ-5** and **TZ-6**.

Crystal Data	TZ-5	TZ-6
Chemical formula	C_10_H_17_N_5_O_2_	C_10_H_14_N_6_O_2_·H_2_O
*M*_r_	239.29	268.29
Crystal system, space group	Triclinic, *P*-1	Triclinic, *P*-1
Temperature (K)	100	100
*a*, *b*, *c* (Å)	10.4130 (11), 11.425 (2), 12.2354 (12)	6.5042 (3), 7.5629 (4), 13.1645 (7)
α, β, γ, (°)	99.703 (7), 114.649 (5), 102.470 (7)	78.729 (3), 85.280 (3), 87.848 (3)
*V* (Å^3^)	1,235.7 (3)	632.77 (6)
*Z*	4	2

#### 3.2.2. Nuclear Magnetic Resonance (NMR)

The ^1^H- and ^13^C-NMR Heteronuclear Single Quantum Coherence (g-HSQC) and Heteronuclear Multiple Bond Correlation (g-HMBC) (1H-13C i 1H-15N) spectra were acquired on the Varian vnmrs 600 spectrometer at 599.94 MHz transmitter frequency for ^1^H at the Institute of Organic Chemistry Polish Academy of Science in Warsaw. The spectra were measured in the DMSO-d_6_ solution at room temperature. The chemical shift standards were as follows: liquid TMS (0.00 ppm for ^1^H-NMR), residual signal of DMSO (39.50 ppm for ^13^C-NMR) and liquid nitromethane (0.00 ppm for ^15^N-NMR).

#### 3.2.3. Infrared Spectroscopy (IR)

The infrared spectra were obtained in the range from 4,000 to 400 cm^−1^ with the spectral resolution of 4 cm^−1^ using a Nicolet iS10 FT-IR spectrometer (Thermo Scientific, Waltham, MA, USA) and KBr pellets.

#### 3.2.4. Raman Spectroscopy

FT Raman spectra were recorded on a Nicolet NXR 9650 instrument (Thermo Scientific), using 1,064 nm excitation from Nd:YVO_4_ laser, in the range from 3,700 to 150 cm^−1^ with a spectral resolution of 8 cm^−1^.

#### 3.2.5. Thermal Analysis (DSC, TGA)

Thermal analyses were carried out on a DSC 822 differential scanning calorimeter equipped with IntraCooler and on a TGA/SDTA 851cells thermogravimeter (Mettler Toledo GmbH, Schwerzenbach, Switzerland) under nitrogen atmosphere. Accurately weighed samples (4–7 mg) were packed in aluminum pans with pierced lids. TGA measurements were blank curve corrected.

#### 3.2.6. Water Content

Water content determination was done by Karl Fischer volumetric titration using the Methrohm 701 KF Titrino apparatus (Methrohm AG, Herisau, Switzerland).

#### 3.2.7. High-Performance Liquid Chromatography (HPLC)

The purity of temozolomide produced in PRI according to an HPLC method [18] is not less than 99.50%. It has been proved experimentally, in a small-plant scale, that in order to obtain such high purity of **TZ-7** in a desired polymorph form III [10], the purity of the intermediate **TZ-6** and the starting material **TZ-5** should not be less than 99.7% and 97.5%, respectively. Lower purity of **TZ-6** leads to a higher level of impurities in **TZ-7** which are difficult to remove during crystallisation. The purification of **TZ-7** leads to its degradation to 5-amino-4-imidazolecarboxamide (AIC) or to the partial transformation, mainly into polymorph IX [10].

The determination of the chemical purity of **TZ-7**, **TZ-6** and **TZ-5** was carried out using Waters Alliance 2695e HPLC systems (Waters, Milford, MA, USA) equipped with gradient pumps, column heaters, autosamplers and either a UV-Vis 2487 or a PDA 2998 detector. The separations during the determination of TZ-7 purity were performed using Aqua C18, 250 × 4.6 mm, 5 µm column (Phenomenex, Torrance, CA, USA). Temozolomide-related substances were separated using water with 0.5% acetic acid and acetonitrile as Eluent A and Eluent B, respectively. The following gradient elution program was applied, t (min)/%B: 0/8; 8/8; 28/40; 31/8; 36/8. Other chromatographic parameters were as follows: column temperature: 25 °C; autosampler temperature: 5 ± 2 °C; flow rate: 1 mL min^−1^; injection volume: 20 µL; UV detection wavelength: λ = 254 nm; diluent: water with 0.5% HAc:ACN (4:1, v/v); the concentration of temozolomide in the investigated sample solutions: 1 mg mL^−1^. The chemical purity of **TZ-6** was determined with the use of a XTerra C18, 250 × 4.6 mm, 5 µm column (Waters). 0.1% H_3_PO_4_ and methanol were used as Eluent A and Eluent B, respectively. The following gradient elution program was applied, t (min)/%B: 0/5; 20/50; 30/50; 32/5; 40/5. Other chromatographic parameters were as follows: column temperature: 25 °C; autosampler temperature: 15 °C; flow rate: 1 mL min^−1^; injection volume: 20 µL; UV detection wavelength: λ_1_ = 254 nm, λ_2_ = 201 nm; diluent: water with 0.1% H_3_PO_4_-MeOH (9:1, v/v); the concentration of TZ-6 in the investigated sample solutions: 0.5 mg mL^−1^. The chemical purity of **TZ-5** was investigated using Aqua C18, 250 × 4.6 mm, 5 µm column. The separations were carried out using 0.1% H_3_PO_4 _and methanol as Eluent A and Eluent B, respectively. The following gradient elution program was applied, t (min)/%B: 0/0; 4/0; 15/50; 30/50; 32/0; 40/0. Other chromatographic parameters were as follows: column temperature: 25 °C; autosampler temperature: 5 ± 2 °C; flow rate: 1 mL min^−1^; injection volume: 10 µL; UV detection wavelength: λ_1_ = 254 nm, λ_2_ = 201 nm; diluent: ACN; the concentration of **TZ-5** in the investigated sample solutions: 0.5 mg mL^−1^.

## 4. Conclusions

The structures of **TZ-5** and **TZ-6** were confirmed by X-ray diffraction and spectroscopic methods. The compounds were compared to temozolomide in the structural aspect. The thermal characteristics of the compounds were also presented. **TZ-5** and **TZ-6** were successfully obtained on a validated small-plant scale. The process is well documented as sets of operational instructions, batch reports and validation protocols. Two methods for the purity determination of both compounds were developed. The high purity of **TZ-6** is crucial for obtaining temozolomide in polymorph form III with purity higher than 99.50%.
